# Sigmoid colon perforation presumed to be stercoral in origin in an elderly patient with chronic constipation: a case report

**DOI:** 10.3389/fmed.2026.1872114

**Published:** 2026-07-17

**Authors:** Wenwei Zuo, Jiexin Bai, Fengsheng Yin, Chuan Long

**Affiliations:** 1 Hospital of Chengdu University of Traditional Chinese Medicine, Chengdu, Sichuan, China; 2 Eye College of Chengdu University of Traditional Chinese Medicine, Chengdu, Sichuan, China; 3 Department of Orthopedics, Chengdu Integrated TCM and Western Medicine Hospital, Chengdu University of Traditional Chinese Medicine, Chengdu, Sichuan, China

**Keywords:** case report, chronic constipation, computed tomography, fecaloma, stercoral perforation

## Abstract

Stercoral perforation (SP) is an uncommon but potentially life-threatening complication of chronic constipation that predominantly affects elderly individuals. Early diagnosis is challenging because of nonspecific clinical manifestations and the risk of rapid progression to feculent peritonitis and sepsis. We report the case of an 85-year-old woman who presented with severe acute lower abdominal pain, abdominal distension, nausea, vomiting, and absence of flatus or bowel movements. She had a 6-month history of chronic constipation and long-term antihypertensive therapy with levamlodipine besylate. Computed tomography (CT) demonstrated free intraperitoneal air, abdominal fluid, and fecal impaction within the sigmoid colon. Emergency laparoscopic exploration followed by limited open surgery revealed severe feculent peritonitis and a 2.5 cm × 2.0 cm perforation of the distal sigmoid colon. Necrotic tissue at the perforation margin was debrided, and the perforated sigmoid segment was exteriorized and matured as a colostomy. No bowel resection was performed because diffuse ischemia or necrosis was not identified intraoperatively and the patient was considered high risk because of advanced age and poor baseline condition. The diagnosis of probable stercoral perforation was based on the clinical presentation, radiological findings, and intraoperative observations. Postoperatively, the patient required intensive care monitoring and received broad-spectrum antimicrobial therapy, nutritional support, and electrolyte replacement. She was discharged in stable condition on postoperative day 14. At the 11-week follow-up, she tolerated a regular diet and reported regular bowel movements. At 1-year follow-up, she remained independent in daily activities, with no recurrent perforation, severe constipation, or rehospitalization. This case highlights the importance of early CT evaluation, individualized surgical decision-making, prompt source control, and comprehensive perioperative management in elderly patients with suspected stercoral perforation.

## 1 Introduction

Stercoral perforation (SP) is a rare acute abdominal emergency caused by pressure ischemia and necrosis of the colonic wall due to impacted hardened fecal masses, also known as fecalomas (1). Since its first description in 1894, fewer than 150 confirmed cases of SP have been reported worldwide, and most cases have occurred in elderly patients with chronic constipation, although younger patients and individuals with specific predisposing conditions may also be affected (2).

The pathophysiology of SP is generally attributed to sustained intraluminal pressure from fecalomas, which may exceed capillary perfusion pressure and lead to localized ischemia, mucosal ulceration, transmural necrosis, and eventual perforation. The sigmoid colon and rectosigmoid junction are particularly vulnerable because of high intraluminal pressure, relatively dehydrated stool, and limited blood supply along the antimesenteric border (3, 4). Known risk factors include chronic constipation, advanced age, impaired mobility, neurological or cognitive disorders, and medications that reduce intestinal motility or promote constipation, such as opioids and antipsychotic agents (5). Calcium channel blockers may also contribute to constipation in susceptible patients, although their direct association with SP remains insufficiently documented and requires further clinical evidence.

The clinical presentation of SP is often non-specific, including abdominal pain, abdominal distension, nausea, vomiting, and symptoms of bowel obstruction, which makes early diagnosis difficult. The preoperative diagnosis rate has been reported to be approximately 11%, and delayed recognition may lead to fecal peritonitis, sepsis, and high mortality (6). Computed tomography (CT) plays a pivotal role in early diagnosis by demonstrating fecaloma, free intraperitoneal air, colonic wall discontinuity, pericolonic inflammatory changes, and signs of intra-abdominal contamination.

Herein, we report a case of probable stercoral perforation of the sigmoid colon in an 85-year-old woman with a history of chronic constipation. The diagnosis was based on clinical presentation, radiological findings, and intraoperative observations. This case highlights the importance of early CT evaluation, individualized surgical decision-making, perioperative antimicrobial management, and long-term follow-up in elderly patients presenting with suspected stercoral perforation.

## 2 Case presentation

An 85-year-old woman was admitted with acute lower abdominal pain lasting 4 h. The pain was severe and distending in nature and was accompanied by nausea, two episodes of vomiting, abdominal distension, and absence of flatus or bowel movements. She had no fever or chills. The patient had a 6-month history of chronic constipation and had used glycerin suppositories with poor response. She also had a history of hypertension for more than 30 years and had been taking oral levamlodipine besylate 2.5 mg once daily. Her previous surgical history included cholecystectomy more than 20 years earlier.

On admission, her body temperature was 36.2 °C, heart rate was 96 beats/min, respiratory rate was 23 breaths/min, and blood pressure was 107/66 mmHg. Physical examination revealed lower abdominal tenderness, mild rebound tenderness, muscular guarding, and decreased bowel sounds. Initial laboratory tests showed an elevated C-reactive protein level of 24.61 mg/L and hypokalemia, with a serum potassium level of 3.31 mmol/L. Coagulation-related markers were also elevated, including fibrinogen 4.49 g/L, D-dimer 2.04 μg/mL, and fibrinogen degradation products 7.25 μg/mL. Selected perioperative laboratory findings are summarized in Table 1.

**TABLE 1:** Perioperative trends in selected laboratory parameters during hospitalization.

Variable	POD0	POD1	POD2	POD3	POD4	POD13
WBC, ×10^9^/L	–	2.66	12.17	16.04	11.76	–
CRP, mg/L	24.61	42.21	184.56	154.31	101.7	31.23
PCT, ng/mL	–	34.37	17.39	7.72	–	–
IL-6, pg/mL	–	5044.36	–	30.28	–	–
Lactate, mmol/L	–	1.76	1.26	1.61	1.58	–
Hemoglobin, g/L	–	83	100	97	96	85
Albumin, g/L	–	27.8	32.4	33.8	31.5	27.5
Potassium, mmol/L	3.31	3.99	3.47	3.22	3.48	3.43
Fibrinogen, g/L	4.49	–	–	–	–	–
D-dimer, μg/mL	2.04	3.62	–	4.69	–	–
FDP, μg/mL	7.25	14.17	–	48.25	–	–

POD, postoperative day; WBC, white blood cell count; CRP, C-reactive protein; PCT, procalcitonin; IL-6, interleukin-6; FDP, fibrinogen degradation products. – Indicates that the parameter was not measured on that day.

Imaging studies: Abdominal CT revealed scattered free intraperitoneal air (Figure 1a), mild colonic dilatation, mesenteric and omental edema, small-volume peritoneal and pelvic fluid collections, and increased intestinal contents. A high-density fecal mass was identified within the distal sigmoid colon, consistent with fecal impaction (Figures 1b, c). Taken together, these findings suggested hollow viscus perforation with intra-abdominal contamination, most likely involving the sigmoid colon. The CT findings were subsequently correlated with the intraoperative findings of distal sigmoid colon perforation, impacted fecal material, and feculent peritonitis.

**FIGURE 1:**
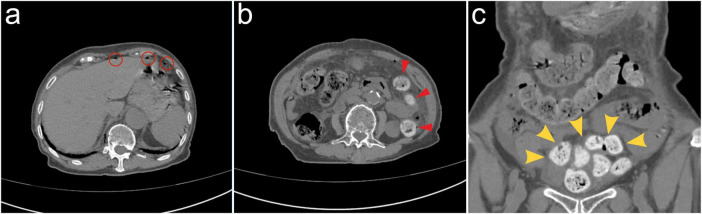
Preoperative abdominal computed tomography (CT) images. **(a)** Axial abdominal CT image showing scattered free intraperitoneal air (red circle). **(b)** Axial abdominal CT image showing increased intestinal contents (red arrow). **(c)** Coronal abdominal CT image showing a high-density lesion in the sigmoid colon, consistent with a fecaloma (yellow arrow).

Surgical intervention: Laparoscopic exploration was performed and revealed severe feculent peritoneal contamination. The operation was converted to limited open surgery. Intraoperatively, a 2.5 cm × 2.0 cm perforation was identified in the distal sigmoid colon, with adherent fecal material and purulent debris (Figure 2). Localized necrotic tissue at the perforation margin was debrided. The remaining inspected colon appeared viable, without diffuse ischemia or necrosis. No obvious diverticular disease, colonic mass, inflammatory bowel disease-related lesion, or other gross pathological abnormality that could account for the perforation was identified during intraoperative exploration. No bowel resection was performed. The sigmoid segment containing the perforation was exteriorized through the abdominal wall, and the debrided perforation was matured directly as a colostomy. Abdominal drains were placed. The operative time was approximately 2 h. Based on the clinical presentation, CT findings, and intraoperative findings, the diagnosis was considered probable stercoral perforation of the sigmoid colon complicated by feculent peritonitis. No histopathological confirmation was available.

**FIGURE 2:**
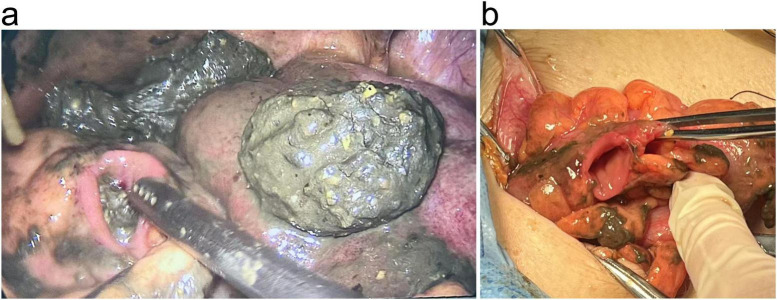
Intraoperative findings of stercoral perforation. **(a)** Laparoscopic exploration revealed fecaloma and a large amount of feculent ascites in the abdominal cavity. **(b)** Open laparotomy revealed an approximately 2.5 cm × 2 cm perforation in the distal sigmoid colon.

Postoperative cardiology consultation was obtained. The consultation suggested that the patient’s chronic constipation might have been associated with long-term use of levamlodipine besylate. Therefore, her antihypertensive regimen was changed to valsartan 80 mg once daily to reduce the risk of medication-induced constipation.

Postoperatively, the patient was transferred to the intensive care unit on postoperative day 1 for close monitoring and supportive treatment. Laboratory tests showed marked systemic inflammation, including procalcitonin 34.37 ng/mL and interleukin-6 5044.36 pg/mL, while serum lactate was 1.76 mmol/L. Antimicrobial therapy with imipenem and tigecycline was initiated. On postoperative day 2, the patient was transferred back to the Department of General Surgery. Serum β-D-glucan was <10 pg/mL. Cefoperazone-sulbactam was added on postoperative day 4. Blood cultures obtained on postoperative day 6 showed no growth. Imipenem and tigecycline were discontinued on postoperative day 8, and cefoperazone-sulbactam was continued alone. After clinical and laboratory reassessment, all antibacterial therapy was discontinued on postoperative day 13.

During hospitalization, electrolyte replacement and nutritional support were provided for hypokalemia, hypophosphatemia, and hypoalbuminemia. On postoperative day 13, the patient was conscious and clinically stable, with no abnormal discharge from the stoma. Laboratory tests showed residual anemia and inflammatory response, including hemoglobin 85 g/L and C-reactive protein 31.23 mg/L. Serum potassium was 3.43 mmol/L and albumin was 27.5 g/L. The patient was discharged in stable condition on postoperative day 14.

At the 11-week follow-up, she tolerated a regular diet, had regular bowel movements through the colostomy, and reported no abdominal pain or fever. Her general condition had improved. At the 1-year follow-up, she remained independent in daily activities, with no recurrent perforation, severe constipation, or rehospitalization. The overall clinical course is summarized in Figure 3.

**FIGURE 3:**
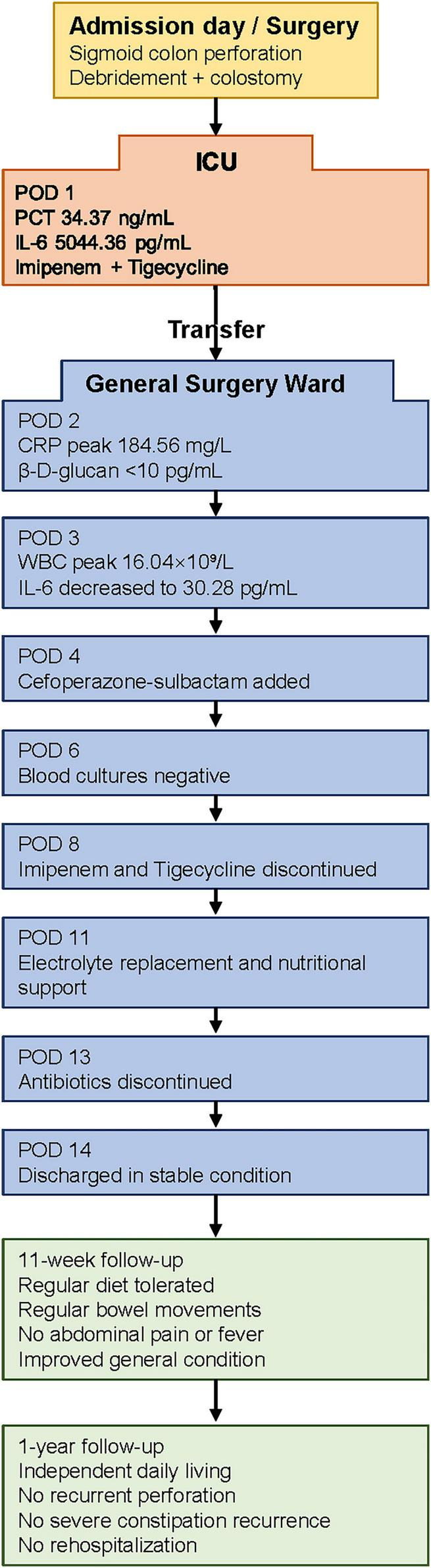
Clinical timeline of surgical treatment, postoperative course, and follow-up. ICU, intensive care unit; POD, postoperative day; CRP, C-reactive protein; WBC, white blood cell count; PCT, procalcitonin; IL-6, interleukin-6.

## 3 Discussion

The diagnosis in this case was based on a history of chronic constipation, CT findings of fecal impaction and free intraperitoneal air, and intraoperative findings of feculent peritonitis, impacted fecal material, and sigmoid colon perforation. These findings are consistent with the commonly described clinical and operative characteristics of stercoral perforation reported in previous studies (7–9). In addition, no obvious diverticular disease, colonic mass, inflammatory bowel disease-related lesion, or other gross pathological abnormality was identified during intraoperative exploration. However, no bowel resection or tissue biopsy was performed; therefore, the characteristic histopathological features of stercoral perforation, including pressure ulceration and transmural necrosis, could not be confirmed. Consequently, although the clinical, radiological, and intraoperative findings were compatible with a stercoral etiology, the diagnosis should be regarded as probable stercoral perforation rather than a histologically confirmed case.

Computed tomography provided important information for diagnosis and operative planning in the present case. Because the clinical presentation is often nonspecific, differentiation from bowel obstruction, diverticular perforation, ischemic colitis, or other causes of acute abdomen may be difficult (10, 11). In the present patient, CT demonstrated fecal impaction, free intraperitoneal air, and findings suggestive of intra-abdominal contamination, all of which were subsequently confirmed intraoperatively. Consistent with previous reports, CT provided important information for both diagnosis and operative planning (12, 13).

Most published reports recommend resection of the perforated and diseased bowel segment because residual diseased colon may increase the risk of recurrent perforation (9, 14, 15). Hartmann’s procedure is among the most frequently reported surgical approaches in patients with severe contamination and generalized peritonitis (16, 17). However, our patient was 85 years old and had sepsis, hypoalbuminemia, and poor nutritional status, all of which increased operative risk. In the present case, bowel resection was not performed because of the patient’s advanced age, frailty, and the desire to minimize operative duration and physiological stress. Intraoperatively, necrosis was limited to the perforation margin, whereas the remaining inspected colon appeared viable without diffuse ischemia or necrosis. Therefore, after debridement, contamination control, and abdominal drainage, the perforated sigmoid segment was exteriorized and matured as a colostomy without bowel resection. Therefore, the surgical strategy in this case was selected according to the patient’s individual clinical circumstances rather than as a predefined standard approach for stercoral perforation. Although this approach differs from the resection-based procedures more commonly described in the literature, the favorable outcome observed in this patient demonstrates the clinical course following this individualized management strategy.

In addition to surgical source control, comprehensive perioperative management played an important role in recovery. The patient required postoperative ICU monitoring and demonstrated marked elevations of inflammatory biomarkers, including PCT and IL-6, which gradually decreased during treatment. Broad-spectrum antimicrobial therapy, electrolyte replacement, and nutritional support were provided throughout hospitalization. Similar to previous reports, favorable outcomes were achieved through the combination of timely source control and multidisciplinary perioperative management (7, 18, 19). The patient was discharged on postoperative day 14 and remained free of recurrent perforation or disease-related rehospitalization at both the 11-week and 1-year follow-up visits.

Although the patient had long-term exposure to levamlodipine besylate, the present case does not establish a causal relationship between this medication and stercoral perforation. Advanced age and chronic constipation are well-recognized risk factors and may adequately explain the clinical presentation (5, 7, 20). Nevertheless, medications that may impair bowel motility should be considered when evaluating patients with chronic constipation. Therefore, this observation should be regarded as hypothesis-generating only and warrants further investigation rather than implying causality.

## 4 Conclusion

In this case, probable stercoral perforation was associated with chronic constipation, acute abdominal pain, and feculent peritonitis in an elderly patient. CT findings were useful for preoperative assessment and surgical planning. The patient’s management involved surgical source control, postoperative intensive monitoring, antimicrobial therapy, nutritional support, and long-term follow-up. The present report documents the diagnostic process, operative decision-making, perioperative management, and long-term outcome of an elderly high-risk patient with probable stercoral perforation.

## Data Availability

The original contributions presented in this study are included in this article/supplementary material, further inquiries can be directed to the corresponding author.
